# Decomposing Gender Disparity in Total Physical Activity among Iranian Adults

**DOI:** 10.4178/epih.e2017044

**Published:** 2017-10-16

**Authors:** Ebrahim Rahimi, Seyed Saeed Hashemi-Nazari, Koorosh Etemad, Hamid Soori

**Affiliations:** 1 Department of Epidemiology, School of Public Health, Shahid Beheshti University of Medical Sciences, Tehran, Iran; 2 Safety Promotion and Injury Prevention Research Center, School of Public Health, Shahid Beheshti University of Medical Sciences, Tehran, Iran

**Keywords:** Gender identity, Exercise, Physical exertion, Socioeconomic factors

## Abstract

**OBJECTIVES:**

While gender differences in physical activity (PA) have been reported, their origin is not well understood. The present study aimed to identify factors contributing to this disparity.

**METHODS:**

This was a population-based cross-sectional study based on the 2011 surveillance of risk factors of non-communicable diseases that was conducted among Iranian adults. Multi-staged sampling was performed to obtain the required study sample. The primary outcome was gender differences in the prevalence of sufficient physical activity (SPA). Total physical activity (TPA) was calculated as metabolic equivalents (MET) per minute during a typical week, as recommended by the World Health Organization. On this basis, achieving 600 MET-min/wk or more was defined as SPA. The nonlinear Blinder-Oaxaca decomposition technique was used to explain the disparity.

**RESULTS:**

The predicted gap was 19.50%. About one-third of the gap was due to differences in the level of observable covariates. Among them, work status contributed the most (29.61%). A substantial portion of the gap remained unexplained by such differences, of which about 40.41% was related to unobservable variables. The differential effects of standard of living, ethnicity, and smoking status made the largest contribution, accounting for 37.36, 35.47, and 28.50%, respectively.

**CONCLUSIONS:**

Interventions to reduce the gender gap in PA should focus on increasing TPA among housewives and women with chronic diseases, as well as those with a higher standard of living. In addition, it is essential to explore the impact of ethnicity and smoking status on women’s TPA in order to promote health.

## INTRODUCTION

Physical activity (PA) is a major contributor to the prevention of non-communicable diseases (NCDs). It is also associated with other health conditions throughout the world, including obesity, mental health, and mortality. It has been shown that people who are PA have lower mortality rates than those who are inactive [1-3].

The health benefits of increasing the level of PA can be obtained in various ways and do not always include exercise. The largest limitation in some relevant studies is that PA at work, during transportation, and during leisure time has not been considered [3]. To gain a better understanding of PA patterns, assessing individuals’ total physical activity (TPA) in all domains is necessary.

As suggested by the World Health Organization (WHO), adults should perform PA for at least 75 minutes at a vigorous intensity, 150 minutes at a moderate intensity, or an equivalent combination of moderate and vigorous-intensity activity, achieving at least 600 metabolic equivalents (METs) per minute in a typical week. Despite this, few adults regularly participate in PA to promote health; fewer than one-third of adults (over 18 years old) meet the WHO’s recommended levels of PA [4].

To promote PA, it is necessary to obtain information on its prevalence and determinants in the population. Many factors are associated with PA, including age, health status, and the social-physical environment, especially aspects of the environment outside the health system, such as public transportation [5].

In addition, it is also necessary to identify subgroups of the population with low levels of PA for further interventions and preventive measures. Studies conducted in many countries have shown a gender difference in PA. They have often reported less PA among women than among men [4,6-8]. In Iran, some reports using data from the national surveillance of risk factors for NCDs (SuRFNCD) likewise indicated that Iranian adults, particularly women, had low levels of PA as well [6,9]. Based on the 2011 SuRFNCD, Koohpayehzadeh et al. [6] stated that about 28.7% of men and 49.7% of women were classified as having low levels of PA. Since sedentary people are more likely than active people to develop NCDs, this may translate to an increased risk among women. Nevertheless, few studies have assessed the factors that contribute to gender differences in PA.

While gender differences in on the level of contributors might cause disparities in PA, it is also possible that these factors have a differential association with PA in men and women. For example, the effects of equivalent education on PA may differ by gender due to sociocultural factors. Therefore, it is important to clarify whether gender disparities in PA are explained by differences in the level of contributors or the differential effects of the contributing factors between men and women.

However, little is known about the factors contributing to gender disparities in TPA among adults in Iran. Such information is necessary for formulating policies to address this gap.

The aims of the present study were to identify factors associated with a higher level of TPA in men and to estimate how much of the gender difference in PA can be attributed to each of these factors. Therefore, we examined men-women disparities in TPA prevalence in Iranian adults using an extension of the Blinder-Oaxaca decomposition method [10-13] to determine the factors contributing to the gap. To our knowledge, this is the first study to provide detailed information on this subject using this method. It may have policy implications, especially concerning modifiable factors [14].

The Blinder-Oaxaca method, an approach for decomposing inequality used in economics [11,12], has proven useful for understanding the underlying causes of disparities in health outcomes [14-23]. The original method used a counterfactual regression approach decomposing the difference in a mean outcome of interest between 2 groups into a part that is explained by group differences in levels of observable characteristics and a part attributable to the differential effect of these characteristics between 2 groups and the effect of the other unknown factors that were not included in the model. Thus, the method quantifies the contributions from different parts [10-12].

## MATERIALS AND METHODS

The study data were obtained from the SuRFNCD-2011 of Iranian adults. This was a population-based cross-sectional study based on the WHO’s STEPwise approach to NCD risk factor surveillance (STEPS) [24,25]. The survey used a multi-stage sampling method to obtain the required study sample. Details of the survey can be found elsewhere [6,26].

The primary outcome was a difference in the prevalence of sufficient physical activity (SPA) between men and women. TPA was calculated as METs per minute during a typical week using the Global Physical Activity Questionnaire as suggested by the WHO. On this basis, achieving 600 MET-min/wk or more was defined as SPA. The questionnaire collected information on PA in 3 domains, including PA at work, during transportation (walking and/or cycling), and during leisure time, and covered various components of PA, such as frequency, duration, and intensity [27,28].

A set of covariates that could potentially affect PA behavior was categorized into predisposing, enabling, and need-related factors based on Andersen’s socio-behavioral model [29].

Age (years), family size, educational status (less than high school, a high school diploma, or at least some college), work status, and ethnicity (Fars or other) were considered predisposing factors [30, 31]. Work status was defined based on the Bureau of Labor Statistics. Subjects who had no job and were not looking for work were counted as not in the labor force. This category included housewives, students, and those unable to work [32].

The covariates categorized as enabling factors were standard of living, geographical region (province of current residence), and rural/urban residence [30,31]. Results for the sets of covariates, including living area (m^2^) and the presence of some housing amenities, such as a vacuum cleaner, personal computer, separate freezer, washing machine, and sole use of the bathrooms and kitchen, were considered part of a household’s standard of living. To distinguish between rural and urban residences, every geographical area with a municipality was considered a city (urban area) according to the Statistical Center of Iran.

The covariates identified as need-related factors were the subjects’ history of chronic conditions (including cardio/cerebrovascular disease, osteoporosis, asthma, colon cancer, and type 2 diabetes), high blood pressure (> 140/90 mmHg), smoking status, and overweight/obese (body mass index of 25.0 kg/m^2^ or higher) [30].

Gender differences in covariates were examined using the chisquare test and the Student *t*-test. Multiple logistic regression analysis was performed with MET-minutes as the dependent variable for all subjects. An extension of the Blinder-Oaxaca decomposition for nonlinear models was conducted to explain gender disparities in proportion to SPA. Because the decomposition estimates are sensitive to the choice of a reference group [10,33], in our case, the men group with the higher prevalence of SPA was preferred as the reference. Accordingly, the regression coefficients from the equation for men were used as the reference coefficients.

The application of the original (linear) method to nonlinear decomposition models had also other conceptual problems that affected the results. One of them is known as the identification problem, in essence, the decomposition estimates for categorical variables also depended on the selection of the baseline (omitted) category [10,34]. We handled this issue by computing the decomposition based on “normalized” effects, as proposed by Yun [35]. This method was equivalent to averaging the coefficient effects of a set of dummy variables representing a categorical variable while changing the baseline groups [34].

Another issue was path dependency, in which the contribution of each covariate to the gap depends on the values of the other covariates and also the order in which those enter the decomposition [36]. We employed a convenient solution to this problem, using weights as suggested by Yun [13], so that any ordering of the way that variables enter the decomposition produced the same results due to the weights. The decomposition approach used here is described in detail in the Appendix 1.

All analyses were performed using Stata version 12 (StataCorp., College Station, TX, USA). The decomposition analysis was implemented using an updated Oaxaca package proposed by Yun [13] that supported nonlinear decomposition for binary dependent variables. The package included methods to handle the path dependency and identification problems.

## RESULTS

Table 1 provides descriptive statistics for all the subjects. It also presents a comparison of the level of covariates between gender groups. There were 10,356 adults included in the analyses, of whom 4,289 (41.41%) were men. Approximately 58.10% achieved the WHO’s recommendation for PA (600 MET-minutes or more). About half of the women met the recommendation, whereas about 70.98% of men did so, which was significantly higher than that for women (p< 0.001). Statistically significant gender differences were also found in the levels of all covariates, except for ethnicity (*p*= 0.43) and having a bathroom (*p*= 0.31).

Table 2 shows the adjusted association of each covariate with the level of PA for men and women separately.

In women, the household’s standard of living, educational status, the presence of hypertension, and history of chronic disease were significantly associated with PA (p< 0.05), and only educational status had positive associations.

Among men, only age, work status, and history of chronic disease had statistically significant relationships with the prevalence of PA, and all were inversely associated.

Furthermore, some covariates such as smoking status were associated with PA differently in each gender. Current smoking was positively associated with PA in women, whereas in men the association was inverse. However, both relationships were statistically insignificant.

Table 3 presents the decomposition results for gender differentials in the level of PA. As shown, differences in the level of observed covariates accounted for about one-third of the overall gap (the explained component). Equalizing the level of all covariates across the gender groups could potentially reduce the gap by 0.065 percentage points (33.33%= -0.065/-0.195). The substantial portion of the residual part of the gap (38.59%) that could not be explained by such differences was due to gender differences in unobservable (unknown) covariates, which were not included in the model (the constant residual part), and the rest was attributed to the differential effect of the observed covariates.

In terms of more details on the endowments effect, differences in the level of predisposing factors made a significant contribution to the PA gap, accounting for about 30.48%. Among the predisposing factors, work status contributed the most to the gap. This indicates that the gender difference in proportion to this variable (Table 1) accounted for a reduction (as indicated by the positive sign) in the gap by 29.61%. The gender difference in proportion to the history of chronic disease from need-related factors (33.59% in women vs. 22.17% in men) also contributed significantly to the gap, accounting for about 4.55%.

As shown in detail, the differential effects of enabling and need-related factors explained 30.65 and 29.09% of the PA disparity (with respect to the positive sign); however, they were not statistically significant. Among these factors, the standard of living and smoking status made the largest contribution to the disparity. Despite the negative contribution of the predisposing factors, the differential effects of age and ethnicity (Table 2) contributed significantly to the gap, accounting for -78.67 and 35.47%, respectively. However, eliminating the differential effects of ethnicity alone could reduce the gap.

## DISCUSSION

A nonlinear extension of the Blinder-Oaxaca decomposition method was used to assess the gender difference in SPA prevalence and to determine its contributing factors. A significant gender difference in PA was found. The level of PA in women was far lower than that of men, as defined according to the recommendations of the WHO for health [37,38]. This finding is consistent with the majority of previous reports in this field [4-8,39].

A small portion of the gap could be explained by the heterogeneous distribution of common factors associated with PA behavior. These factors are defined in line with Andersen’s socio-behavioral model model [29] involving 3 sets of related factors, including the factors that have the potential to influence PA behavior— either by encouraging it to occur (predisposing factors) or by inhibiting the behavior from occurring (enabling factors)—and the factors concerning the individual’s perceived need for PA (need-related factors).

According to the findings of this study, the predisposing factors, in particular work status, accounted for the largest contribution to the gender difference in SPA prevalence. This finding is not too far-fetched for 2 reasons: (1) a significant percentage of women are not involved in the labor force [40] and live traditionally as housewives [41]; and (2) the jobs assigned to women demand less PA than those of men. In addition to different work status, the different distribution of the history of chronic diseases in the gender groups plays a somewhat significant role. Chronic diseases can affect PA behavior. In addition, though, it should be noted that a sedentary lifestyle can be a risk factor for chronic diseases, meaning that a two-way relationship must be considered.

As the findings suggest, a substantial portion of gender differences in PA remained unexplained by the uneven distributions of the above factors between the genders. This component accounted for different relationships or associations between these factors and PA in each group, as well as the effects of other unknown factors. The contribution of unknown factors to the PA gap was relatively high. Therefore, future research is strongly recommended to identify and assess the contribution of each unknown factor, along with the other causes involved.

In the unexplained part, the differential effect of enabling factors, particularly standard of living, made a substantial contribution to the disparity. In both men and women, PA decreased as the standard of living improved. As shown in Table 2, this trend was stronger among women than among men.

According to our findings, smoking status was related to PA differently for men than for women, which was one of the reasons for the gender difference in PA. This difference suggests that behavioral responses to smoking may differ between genders due to factors including personality, perceived health risks and benefits, and socioeconomic status [42]. The different distribution of these parameters among men and women may help explain the gender difference in PA. A careful appraisal of this phenomenon may be applied to reduce the PA gap. Thus, investigating the gender difference in the associations between smoking and PA in more detail remains a task for further research.

Among the predisposing factors, the differential effect of ethnicity reduced the gender difference in PA to a remarkable extent. This finding is consistent with the fact that ethnicity plays a major role in shaping traditional attitudes toward women and building a model of traditional family life (wife/mother role), which has restricted working and PA for this segment of society. Because of their ethnic traditions, women experience more difficult and unfair conditions than men, meaning that they are less likely to engage in PA [43]. Therefore, it would be appropriate to conduct another study specifically on the relationships among gender, ethnicity, and PA to identify the informal mechanisms leading to health inequality.

This was the first research project in which the Blinder-Oaxaca decomposition technique was adopted to assess the factors contributing to gender differences in PA. It is also novel in that we used data from the WHO’s STEPS. The survey that was the basis of this study was conducted among a representative sample of adults from 31 provinces of Iran and provided comprehensive information on the NCD risk factors of individuals.

Given its design, this paper attempted to identify statistical associations (rather than causal effects) between the observed covariates and gender differences in SPA prevalence. Therefore, we caution against imputing a causal interpretation of our models.

We conclude that Blinder-Oaxaca decomposition can provide deeper insights into all aspects of disparities, thus paving the way toward equality. According to the findings of this research project, it could be argued that interventions to reduce the gap should focus on promoting PA among housewives and women with chronic diseases, as well as in those with higher standards of living. In addition, it is essential to explore the impact of ethnicity and smoking status on women’s PA in order to promote health.

## Figures and Tables

**Table 1.** Descriptive statistics by gender for all subjects

	All subjects	Women	Men	p-value
Total (n)	10,356	6,067	4,289	-
Sufficient physical activity^1^	58.10	49.13	70.98	<0.001
Predisposing factors				
Age (yr)	42.04± 0.16	42.32±0.20	41.65 ±0.24	0.03
Family size (n)	3.94± 0.02	3.81± 0.02	4.12±0.03	<0.001
Educational status				<0.001
Illiterate	24.93	31.62	15.34	
<High school graduate	36.54	33.71	40.59	
High school graduate	24.36	22.51	27.02	
At least some college	14.17	12.16	17.05	
Work status				<0.001
Employed	31.42	8.99	63.66	
Unemployed	4.64	2.98	7.02	
Non-labor force^2^	63.94	88.03	29.32	
Ethnicity (1 = Fars, 0 = other^3^)	48.69	48.36	49.15	0.43
Enabling factors				
Building area (m^2^)				0.005
<100	47.16	48.46	45.32	-
100-200	42.55	41.75	43.67	-
>200	10.29	9.79	11.00	-
Housing amenities				
Bathroom	93.01	92.80	93.32	0.31
Kitchen	91.88	91.41	92.55	0.04
Vacuum cleaner	84.96	84.35	85.84	0.04
Computer	43.92	40.27	49.18	<0.001
Separate freezer	57.65	55.83	60.26	<0.001
Washing machine	74.89	73.16	77.38	<0.001
Urban residence	70.15	68.68	71.60	0.001
Need-related factors				
Current smoking	9.92	1.19	22.46	<0.001
Overweight/obese^4^	55.35	59.60	49.22	<0.001
Hypertension	29.71	31.36	27.35	<0.001
History of related chronic disease^5^	29.02	33.59	22.17	<0.001

Values are presented as mean±standard deviation or %.

1 Achieving 600 metabolic equivalents-min/wk or more as suggested by the World Health Organization.

2 Subjects who had no job and were not looking for work were counted as not in the labor force according to the Bureau of Labor Statistics. This category included housewives, students, and those unable to work.

3 Including Turk, Lor, Arab, Kurd, Gilak, Turkman, Baluch, Sistani, and others.

4 If the body mass index was 25.0 kg/m^2^ or higher.

5 Having a history of one or more chronic diseases, including cardio/cerebrovascular disease, osteoporosis, asthma, colon cancer, and/or type 2 diabetes.

**Table 2.** Associations of the observed covariates with total physical activity using a multivariable logistic regression model in men and women separately

	Women	p-value	Men	p-value
Age in years	1.004 (0.997, 1.010)	0.26	0.990 (0.982, 0.997)	0.007
Family size	1.037 (0.997, 1.078)	0.07	1.050 (0.999, 1.103)	0.06
Educational status^1^				
< High school graduate	1.316 (1.110, 1.561)	0.002	0.909 (0.713, 1.158)	0.44
High school graduate	1.393 (1.107, 1.754)	0.005	1.019 (0.755, 1.377)	0.90
At least some college	1.417 (1.056, 1.900)	0.02	1.061 (0.757, 1.487)	0.73
Work status^2^				
Unemployed	0.627 (0.367, 1.070)	0.09	0.368 (0.265, 0.512)	<0.001
Non-labor force	0.792 (0.631, 0.994)	0.04	0.606 (0.498, 0.738)	<0.001
Ethnicity^3^	0.849 (0.713, 1.011)	0.07	1.034 (0.829, 1.288)	0.77
Standard of living^4^	0.611 (0.446, 0.838)	0.002	0.889 (0.576, 1.370)	0.59
Urban residence	0.903 (0.774, 1.054)	0.19	0.901 (0.728, 1.114)	0.33
Current smoking	1.514 (0.880, 2.604)	0.13	0.937 (0.779, 1.127)	0.49
Overweight/obese^5^	0.923 (0.806, 1.055)	0.24	0.860 (0.728, 1.016)	0.08
Hypertension	0.866 (0.751, 0.998)	0.04	1.000 (0.833, 1.200)	0.99
History of a chronic condition^6^	0.858 (0.744, 0.990)	0.04	0.687 (0.569, 0.830)	<0.001
Constant	1.936 (1.092, 3.431)	0.02	8.169 (3.973, 16.798)	<0.001

Values are presented as odds ratio (95% confidence interval).

1 The lowest category, illiterate, was used as the reference category.

2 The category of employed, was used as the reference category.

3 Ethnicity: 1, Fars; 0, other ethnic groups including Turk, Lor, Arab, Kurd, Gilak, Turkman, Baluch, Sistani, and others.

4 Results for sets of predictors, including living area (m^2^) and having some housing amenities, were subsumed in proxy measures of the standard of living.

5 If the body mass index was 25.0 kg/m^2^ or higher.

6 Having a history of one or more chronic diseases, including cardio/cerebrovascular disease, osteoporosis, asthma, colon cancer, and type 2 diabetes.

**Table 3.** Detailed decomposition of gender disparity in proportion to SPA^1^ using a nonlinear extension of the Blinder-Oaxaca decomposition method

	Coefficient	95% CI		p-value	Contribution (%)^2^
		LL	UL		
Total difference	-0.195	-0.217	-0.173	<0.001	100.00
Endowments effect^3^	-0.065	-0.097	-0.033	<0.001	33.34
Unexplained effect^4^	-0.130	-0.168	-0.091	<0.001	66.66
Detailed decomposition: endowments effect					
Predisposing factors	-0.059	-0.090	-0.029	<0.001	30.48
Age in years	0.001	-0.001	0.002	0.49	-0.26
Family size	-0.003	-0.007	0.000	0.05	1.69
Educational status^5^	0.001	-0.009	0.011	0.89	-0.33
Work status^6^	-0.058	-0.084	-0.031	<0.001	29.61
Ethnicity^7^	0.000	-0.002	0.003	0.68	-0.22
Enabling factors	0.003	-0.003	0.009	0.349	-1.56
Standard of living^8^	0.000	-0.004	0.004	0.89	-0.13
Urban/rural residence	0.001	-0.001	0.002	0.36	-0.30
Province of current residence	0.002	-0.003	0.007	0.41	-1.12
Need-related factors	-0.009	-0.020	0.002	0.11	4.52
Smoking status^9^	0.004	-0.006	0.013	0.47	-1.85
Overweight/obese^10^	-0.004	-0.008	0.000	0.08	1.83
Hypertension	0.000	-0.002	0.002	0.97	-0.02
History of chronic disease^11^	-0.009	-0.014	-0.004	<0.001	4.55
Detailed decomposition, unexplained effect					
Predisposing factors	0.062	-0.064	0.188	0.34	-31.76
Age in years	0.153	0.048	0.259	0.004	-78.67
Family size	-0.013	-0.067	0.041	0.64	6.68
Educational status^5^	-0.005	-0.022	0.011	0.51	2.75
Work status^6^	-0.004	-0.056	0.048	0.88	2.01
Ethnicity^7^	-0.069	-0.109	-0.030	0.001	35.47
Enabling factors	-0.060	-0.174	0.055	0.34	30.65
Standard of living^8^	-0.073	-0.186	0.041	0.21	37.36
Urban/rural residence	0.001	-0.011	0.012	0.93	-0.27
Province of current residence	0.013	-0.005	0.030	0.16	-6.44
Need-related factors	-0.057	-0.122	0.009	0.09	29.09
Smoking status^9^	-0.056	-0.120	0.009	0.09	28.50
Overweight/obese^10^	0.003	-0.005	0.011	0.49	-1.45
Hypertension	0.004	-0.003	0.011	0.22	-2.24
History of chronic disease^11^	-0.008	-0.017	0.001	0.07	4.27
Constant	-0.075	-0.251	0.101	0.40	38.59

SPA, sufficient physical activity; CI, confidence interval; LL, lower limit; UL, upper limit.

1 Achieving 600 metabolic equivalents-min/wk or above as suggested by the Wordl Health Organization.

2 A negative number implies that reducing the gender difference in the variable widens the gap.

3 Includes the difference in the level of characteristics between men and women.

4 Includes the differential association of characteristics in each group along with a constant.

5 Educational status: 1, illiterate; 2, less than high school graduate; 3, high school graduate; 4, at least some college.

6 Work status: 1, employed; 2, unemployed; 3, if not considered to be in the labor force according to the Bureau of Labor Statistics.

7 Ethnicity: 1, Fars; 2, other ethnic groups including Turk, Lor, Arab, Kurd, Gilak, Turkman, Baluch, or Sistani.

8 Results for sets of predictors, including building area (m^2^) and having some housing amenities were subsumed in proxy measures of standard of living.

9 Smoking status: 1, current smoking; 2, no current smoking.

10 If the body mass index was 25.0 kg/m^2^ or higher.

11 Having a history of one or more chronic diseases, including cardio/cerebrovascular disease, osteoporosis, asthma, colon cancer, and type 2 diabetes.

## Appendix 1.

Decomposition approach using a nonlinear extension of method
