# Supplementary material for: Decomposing Gender Disparity in Total Physical Activity among Iranian Adults
Source: Epidemiol Health. 2017 Oct 16;39:e2017044. doi: 10.4178/epih.e2017044 (PMC5723909; doi:10.4178/epih.e2017044)
Supplement: Supplementary file 1 [file epih-39-e2017044-app1.pdf]

Appendix 1. Decomposition approach using a nonlinear extension of method

Assuming that the outcome value is a function (F) of a linear combination of explanatory variables and associated regression coefficients, $Y = F(\beta X)$. The average difference in the predicted outcome (Y) between men (m) and women (f) can be decomposed as equations (1) and (2):

$$\bar{Y}^m - \bar{Y}^f = [\overline{F(\beta^m X^m)} - \overline{F(\beta^m X^f)}] + [\overline{F(\beta^m X^f)} - \overline{F(\beta^f X^f)}] \quad (1)$$

where Y, X, and β represent an $N \times 1$ outcome vector, an $N \times K$ matrix of explanatory variables, and a $K \times 1$ vector of regression coefficients, respectively. $F()$ refers to any once differentiable function of a linear combination of X to Y. $\overline{F(\beta^m X^f)}$ indicates a counterfactual equation that was constructed by replacing the women' coefficients with those from the men' equation. This is the basis for the counterfactual comparison and therefore the decomposition of the gap. In fact, the gender difference in the average predicted outcome can be decomposed into 2 components by adding and subtracting this counterfactual equation; the first is due to the gender differences in levels of the explanatory variables (the first term on the right-hand side of the equation [1]). This component is called the endowments effect or the explained component. The second component represents the gender effect, which cannot be explained by such differences (the second term on the right-hand side of the equation [1]). In other words, it indicates the gender disparity in the average predicted outcome that would remain if the 2 groups had the same level of explanatory variables. This component also captures the portion of the disparity due to group differences in unobservable (unknown) variables. This component is known as the unexplained component.

An alternative expression for the nonlinear decomposition is:

$$\bar{Y}^m - \bar{Y}^f = [\overline{F(\beta^m X^m)} - \overline{F(\beta^f X^m)}] + [\overline{F(\beta^f X^m)} - \overline{F(\beta^f X^f)}] \quad (2)$$

where the estimated coefficients for the women group are used as the reference coefficients.

Determining the contribution of each variable to each component (the explained and unexplained parts) requires detailed decomposition, which can be performed by sequentially replacing the variable levels/coefficients of one group with those of another group, while keeping other variable levels/covariates constant. This procedure is not a complicated task in the linear decomposition model. Using the nonlinear method, however, it has some conceptual problems that affect the results. One of them is known as the identification problem; in essence, the decomposition estimates for categorical variables depend on the selection of the baseline (omitted) category [1,2]. This issue is handled by computing the decomposition based on the normalized effects, as proposed by Yun [3]. This is equivalent to averaging the coefficients of the effects of a set of dummy variables representing the categorical variable, while changing the baseline groups [2]. Another issue is path dependency; in fact, determining the contribution of each explanatory variable to the differences in outcome between groups depends on the values of the other covariates and also the order in which they are entered into the decomposition [4]. A solution to this issue is to use weights, as suggested by Yun [5], so that any ordering of how variables enter the decomposition produces the same results. Accordingly, the detailed decomposition of equation (1) can be rewritten as equation (3):

$$\bar{Y}^m - \bar{Y}^f = \sum_{k=1}^{k} W^{X_k} [\overline{F(\beta^m x^m)} - \overline{F(\beta^m x^f)}] + \sum_{k=1}^{k} W^{\beta_k} [\overline{F(\beta^m x^f)} - \overline{F(x^f \beta^f)}] \quad (3)$$

where $\sum_{k=1}^{k} W^{X_k} = \sum_{k=1}^{k} W^{\beta_k} = 1$, and $W^{X_k} = \frac{\beta_k^1(\bar{X}_k^1 - \bar{X}_k^2)}{\sum_{k=1}^{k} \beta_k^1(\bar{X}_k^1 - \bar{X}_k^2)}$ and $W^{\beta_k} = \frac{\bar{X}_k^1(\beta_k^1 - \beta_k^2)}{\sum_{k=1}^{k} \bar{X}_k^1(\beta_k^1 - \beta_k^2)}$ indicate the weight of the kth variable in the linearization of the explained and unexplained parts of the gap, respectively [2,5].

Another solution has been proposed by Fairlie [6], which involves randomly ordering the switching distributions. For this purpose, Fairlie suggested a procedure that requires one-to-one matching of individuals from the 2 groups, so there would be an equal sample size in both groups. Otherwise, multiple (e.g., 100 or 1,000 times) random subsamples of one group (the group with a greater sample size) equal to the sample size of the other group are selected and the mean estimate is considered to be the final report.

References

1. Jann B. The Blinder-Oaxaca decomposition for linear regression models. Stata J 2008;8:453-479.
2. Powers DA, Yoshioka H, Yun MS. Mvdcmp: multivariate decomposition for nonlinear response models. Stata J 2011;11:556-576.
3. Yun MS. A simple solution to the identification problem in detailed wage decompositions. Econ Inq 2005;43:766-772.
4. Fairlie RW. Addressing path dependence and incorporating sample weights in the nonlinear Blinder-Oaxaca decomposition technique for logit, probit and other nonlinear models; 2017 [cited 2017 Oct 25]. Available from: https://siepr.stanford.edu/sites/default/files/publications/17-013.pdf.
5. Yun MS. Decomposing differences in the first moment. Econ Lett 2004;82:275-280.
6. Fairlie RW. An extension of the Blinder-Oaxaca decomposition technique to logit and probit models. J Econ Soc Meas 2005;30:305-316.
